# Supplementary material for: Leveraging permutation testing to assess confidence in positive-unlabeled learning applied to high-dimensional biological datasets
Source: BMC Bioinformatics. 2024 Jun 19;25:218. doi: 10.1186/s12859-024-05834-2 (PMC11186207; doi:10.1186/s12859-024-05834-2)
Supplement: Supplementary file 1 — Additional file 1. [file 12859_2024_5834_MOESM1_ESM.pdf]

**Supplemental Tables**	
Supplemental Table 1	Statistics output from multiple comparison methods applied in Figure 2.
Supplemental Table 2	Statistics output from multiple comparison methods applied in Figure 5A.
**Supplemental Figures**	
Supplemental Figure 1	Q-Q plot of z-score p values.

Dataset	Class Separation	Statistic	50% TN		30%TN		10%TN	
			EPR	MBS	EPR	MBS	EPR	MBS
Synthetic datasets	2 (easy)	Mean (SD) - Actual	0.905 (0.027)	0.864 (0.020)	0.846 (0.042)	0.791 (0.025)	0.661 (0.053)	0.628 (0.026)
		Cliff's Delta estimate (95% CI)	1<br>(0.998,1)	1<br>(0.998, 1)	0.994<br>(0.972, 0.999)	0.998<br>(0.987, 1)	0.229<br>(-0.076, 0.495)	0.249<br>(-0.055, 0.51)
		P value - z-score	6.204e-05	3.665e-10	3.839e-04	2.839e-05	0.346	0.304
	1(medium)	Mean (SD) -Actual	0.723 (0.047)	0.704 (0.030)	0.743 (0.045)	0.699 (0.031)	0.646 (0.053)	0.616 (0.026)
		Cliff's Delta estimate (95%CI)	0.726<br>(0.471,0.869)	0.922<br>(0.791, 0.972)	0.832<br>(0.636, 0.927)	0.889<br>(0.71, 0.96)	0.004<br>(-0.281,0.289)	-0.004<br>(-0.299, 0.291)
		P value – z-score	0.08	0.011	0.042	0.022	0.468	0.433
	0 (negative control)	Mean (SD) - Actual	0.653 (0.058)	0.636 (0.038)	0.668 (0.062)	0.627 (0.035)	0.640 (0.055)	0.607 (0.027)
		Cliff's Delta: estimate (95% CI)	0.278<br>(-0.024 0.533)	0.451<br>(0.155, 0.673)	0.169<br>(-0.127, 0.438)	0.231<br>(-0.049, 0.477)	-0.104<br>(-0.381 0.19)	-0.158<br>(-0.432, 0.143)
		P value – z-score	0.356	0.225	0.381	0.370	0.524	0.525
WDBC datasets (400)	NA	Mean (SD) - Actual	0.922 (0.013)	0.9 (0.01)	0.855 (0.034)	0.799(0.022)	0.6 (0.056)	0.577 (0.031)
		Cliff's Delta estimate (95% CI)	1<br>(0.998,1)	1<br>(0.998,1)	1<br>(0.998 1)	1<br>(0.998 1)	0.546<br>(0.259, 0.744)	0.6<br>(0.309, 0.788)
		P value – z-score	1.531e-05	2.613e-11	1.245e-05	5.950e-09	0.171	0.152

Supplementary Table 1: Statistics output from multiple comparison methods applied in Figure 2.

Dataset	N_{KP}	Statistics	Permutation = 30		Permutation = 100		Permutation = 500	
			EPR	MBS	EPR	MBS	EPR	MBS
Lakhashe et al. study dataset	10	Mean (SD) - Permuted	0.49(0.207)	0.509(0.116)	0.544(0.184)	0.541(0.111)	0.574(0.189)	0.558(0.112)
		Cliff's Delta estimate (95% CI)	0.963 (0.814, 0.993)	0.996 (0.974, 0.999)	0.946 (0.879, 0.976)	0.988 (0.951, 0.997)	0.92(0.891, 0.941)	0.969 (0.944, 0.983)
		P value (z-score)	0.025	0.009	0.028	0.014	0.044	0.021
		P value (T test)	1.16e-11	4.5e-14	1.47e-35	3.27e-41	3.23e-135	1.22e-61
	20	Mean (SD) -Permuted	0.623(0.144)	0.599(0.087)	0.642(0.116)	0.609(0.07)	0.652(0.122)	0.616(0.077)
		Cliff's Delta estimate (95%CI)	0.929 (0.723, 0.983)	0.998 (0.987, 1)	0.957 (0.894, 0.983)	0.998 (0.99, 1)	0.935 (0.893, 0.961)	0.982 (0.963, 0.991)
		P value (z-score)	0.033	0.011	0.017	0.004	0.026	0.009
		P value (T test)	1.92e-11	5.46e-14	2.22e-28	2.56e-37	3.46e-24	3.45e-30
	30	Mean (SD) - Permuted	0.694(0.098)	0.646(0.066)	0.691(0.086)	0.651(0.058)	0.682(0.096)	0.645(0.062)
		Cliff's Delta: estimate (95% CI)	0.911 (0.732, 0.972)	1(0.998, 1)	0.938(0.86, 0.973)	0.996(0.983, 0.999)	0.943(0.909, 0.965)	0.998(0.994, 1)
		P value (z-score)	0.036	0.003	0.019	0.001	0.025	0.002
		P value (T test)	3.62e-11	1.62e-16	1.01e-31	1.36e-43	3.43e-31	8.14e-36
	40	Mean (SD) - Permuted	0.716(0.085)	0.676(0.064)	0.691(0.086)	0.651(0.058)	0.724(0.08)	0.685(0.055)
		Cliff's Delta estimate (95% CI)	0.94 (0.729, 0.988)	1 (0.998, 1)	0.948 (0.888, 0.976)	1 (0.999, 1)	0.928 (0.885, 0.956)	0.999 (0.997, 1)
		P value (z-score)	0.032	0.002	0.018	0.0002	0.031	0.0009
		P value (T test)	1.85e-11	1.5e-16	1.03e-35	1.56e-59	8.25e-30	2.07e-45

Supplementary Table 2: Statistics output from multiple comparison methods applied in Figure 5A.

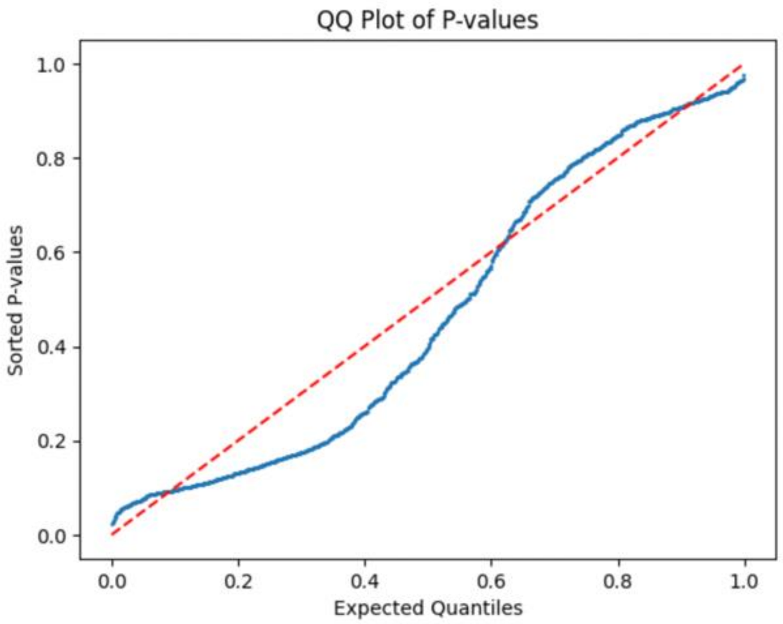

Supplemental Figure 1. Q-Q plot of 1000 replicate calculations of z-test p values (blue) versus the uniform distribution dictated by the null hypothesis (red).
